# Unenhanced computed tomography radiomics help detect endoleaks after endovascular repair of abdominal aortic aneurysm

**DOI:** 10.1007/s00330-023-10000-y

**Published:** 2023-09-02

**Authors:** Ge Hu, Ning Ding, Zhiwei Wang, Zhengyu Jin

**Affiliations:** 1 Medical Research Center, State Key Laboratory of Complex Severe and Rare Disease, Peking Union Medical College Hospital, Chinese Academy of Medical Sciences and Peking Union Medical College, Dongcheng Dist, Beijing, 100730 China; 2 Department of Radiology, State Key Laboratory of Complex Severe and Rare Disease, Peking Union Medical College Hospital, Chinese Academy of Medical Sciences and Peking Union Medical College, Dongcheng Dist, Beijing, 100730 China

**Keywords:** Abdominal aortic aneurysm, Endovascular aneurysm repair, Endoleak detection, Radiomics, Machine learning

## Abstract

**Objectives:**

To explore the feasibility of unenhanced CT images for endoleak detection of abdominal aortic aneurysm (AAA) after endovascular repair (EVAR).

**Methods:**

Patients who visited our hospital after EVAR from July 2014 to September 2021 were retrospectively collected. Two radiologists evaluated the presence or absence of endoleaks using the combination of contrast-enhanced and unenhanced CT as the referenced standard. After segmenting the aneurysm sac of the unenhanced CT, the radiomic features were automatically extracted from the region of interest. Histogram features of patients with and without endoleak were statistically analyzed to explore the differences between the two groups. Twelve common machine learning (ML) models based on radiomic features were constructed to evaluate the performance of endoleak detection with unenhanced CT images.

**Results:**

The study included 216 patients (69 ± 8 years; 191 men) with AAA, including 64 patients with endoleaks. A total of 1955 radiomic features of unenhanced CT were extracted. Compared with patients without endoleak, the aneurysm sac outside the stent of patients with endoleak had higher CT attenuation (41.7 vs. 33.6, *p* < 0.001) with smaller dispersion (51.5 vs. 58.8, *p* < 0.001). The average area under the curve (AUC) of the ML models constructed with unenhanced CT radiomics was 0.86 ± 0.05, the accuracy was 81% ± 4, the sensitivity was 88% ± 10, and the specificity was 78% ± 5. When fixing the sensitivity to > 90% (92% ± 2), the models retained specificity at 72% ± 10.

**Conclusions:**

Unenhanced CT features exhibit significant differences between patients with and without endoleak and can help detect endoleaks in AAA after EVAR with high sensitivity.

**Clinical relevance statement:**

Unenhanced CT radiomics can help provide an alternative method of endoleak detection in patients who have adverse reactions to contrast media. This study further exploits the value of unenhanced CT examinations in the clinical management and surveillance of postoperative abdominal aortic aneurysm.

**Key Points:**

• *Unenhanced CT features of the aneurysm sac outside the stent exhibit significant differences between patients with and without endoleak. The endoleak group showed higher unenhanced CT attenuation (41.7 vs 33.6, p < .001) with smaller dispersion (51.5 vs 58.8, p < .001) than the nonendoleak group.*

• *Unenhanced CT radiomics can help detect endoleaks after intervention. The average area under the curve (AUC) of twelve common machine learning models constructed with unenhanced CT radiomics was 0.86 ± 0.05, the average accuracy was 81% ± 4.*

• *When fixing the sensitivity to > 90% (92% ± 2), the machine learning models retained average specificity at 72% ± 10.*

**Supplementary information:**

The online version contains supplementary material available at 10.1007/s00330-023-10000-y.

## Introduction

In recent years, endovascular aneurysm repair (EVAR) has become the most common treatment for abdominal aortic aneurysm (AAA) [1, 2]. Although EVAR is safer than traditional open repair and is minimally invasive, patients still require long-term follow-up to avoid postinterventional complications [3]. One of the most common complications of EVAR is endoleaks, which signifies the presence of flow in the aneurysm sac outside the stent with an incidence of 30% [3, 4]. Due to the hemodynamic changes in the abdominal aorta, an endoleak can lead to postoperative aneurysm expansion and even rupture [5, 6]. The effective and timely detection of endoleak with appropriate clinical intervention is critical for patients with AAA.

Contrast-enhanced CT, which clearly depicts the anatomic structures of aneurysms and stent grafts, is currently the main reference standard for endoleak detection and surveillance [7]. However, organ-specific reactions, including toxicity associated with renal, cardiovascular, or neurological systems, may occur due to contrast media [8–10]. Therefore, the application of enhanced CT examination in the postoperative follow-up of patients with AAA is limited to some extent by the use of contrast agents.

Due to blood infiltration, the composition of aneurysms should differ between patients with and without endoleak. Contrast media can enhance such differences and provide more exact and precise information regarding the presence and location of endoleaks [8] but cannot change the inherent components of aneurysms. Therefore, differences caused by endoleak should also be reflected on unenhanced CT images, although they are too subtle to be distinguished by human eyes. Compared with contrast-enhanced CT, unenhanced CT is simple and convenient to perform and avoids the need for contrast agent injection. If the image information of unenhanced CT can be fully evaluated, for example, using the methodology of radiomics and machine learning (ML) to extract and analyze image features [11, 12], radiologists may be able to intuitively “see” the differences between aneurysms with or without endoleak in unenhanced CT images.

Radiomics is an emerging image analysis method. High-throughput extraction of quantitative features enables efficient elucidation of subtle characteristics within medical images that cannot be assessed by visual inspection [13, 14]. Previous studies have shown that, based on contrast-enhanced CT, radiomics combined with ML technology can be used to differentiate aggressive from benign endoleaks [15] and predict AAA progression [16] and patient outcomes after EVAR [17]. However, reports based on unenhanced CT images for AAA-related study (especially for endoleak detection) are relatively limited. Although it is difficult to visually “see” endoleaks in unenhanced CT scans, the radiomic features may help radiologists to distinguish the differences between unenhanced CT of patients with and without endoleak.

Based on the above analysis, the purpose of our study is to explore the differences in radiomic features of unenhanced aneurysms between patients with and without endoleak and evaluate the performance of twelve common ML models constructed with unenhanced radiomic features in endoleak detection.

## Materials and methods

### Patient selection

**Fig. 1:**
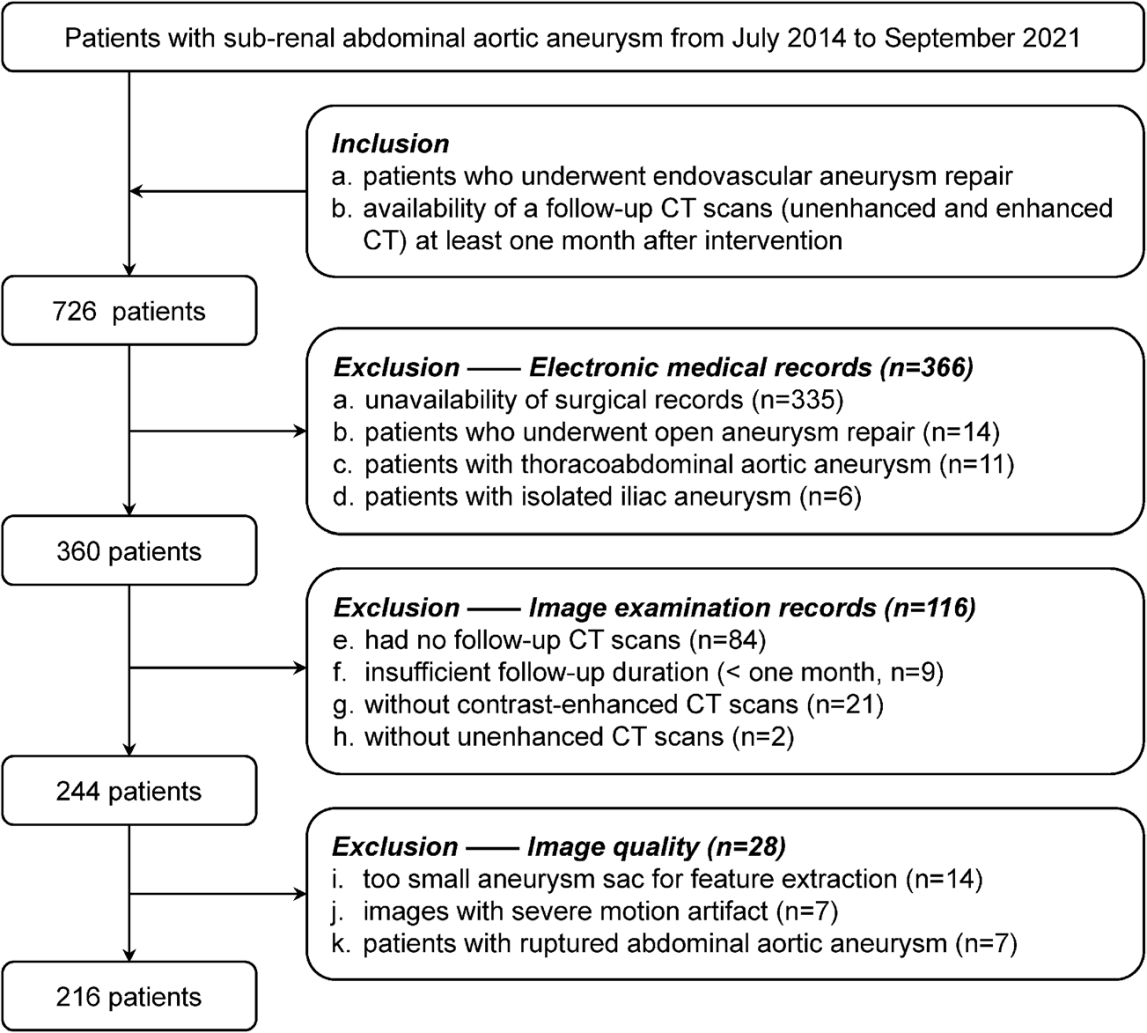
Patient selection flowchart

**Fig. 2:**
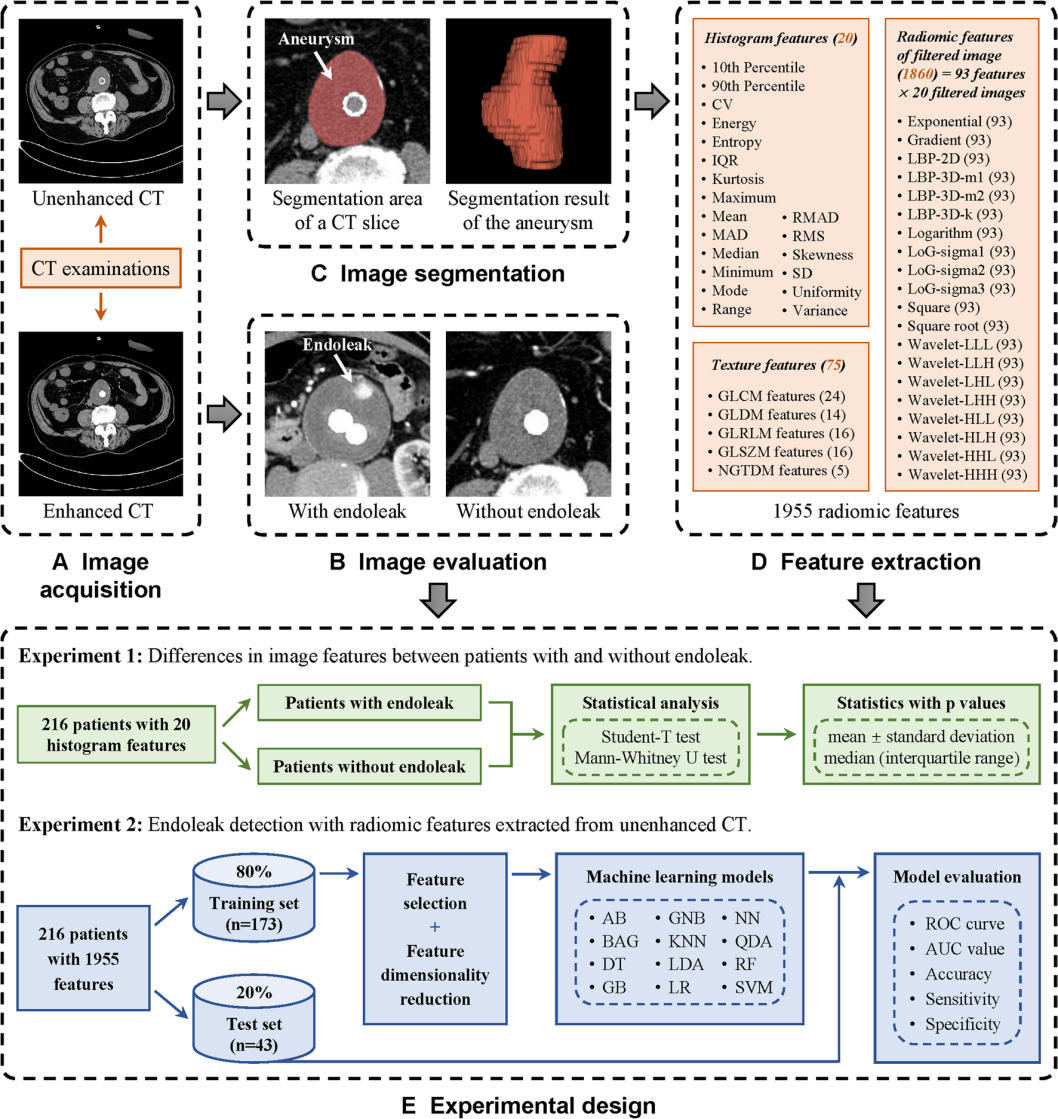
The framework of the study. **A** Image acquisition. **B** Image evaluation. **C** Image segmentation. **D** Feature extraction. **E** Experimental design. CV, coefficient of variation; IQR, interquartile range; MAD, mean absolute deviation; RMAD, robust mean absolute deviation; RMS, root mean squared; SD, standard deviation; GLCM, gray-level co-occurrence matrix; GLDM, gray-level dependence matrix; GLRLM, gray-level run-length matrix; GLSZM, gray-level size zone matrix; NGTDM, neighboring gray-tone difference matrix; LBP, local binary pattern; LoG, Laplacian of Gaussian; AB, adaptive boosting; BAG, bagging; DT, decision tree; GB, gradient boosting; GNB, Gaussian Naive Bayes; KNN, *k*-nearest neighbors; LDA, linear discriminant analysis; LR, logistic regression; NN, neural network; QDA, quadratic discriminant analysis; RF, random forest; SVM, support vector machine; ROC, receiver operating characteristic; AUC, area under the curve

The institutional review board of Peking Union Medical College Hospital approved this retrospective study and waived the requirement for informed consent. We initially collected 726 patients with AAA who visited our hospital from July 2014 (the earliest date that can be retrieved in the system) to September 2021 (Fig. 1) by querying the electronic medical record (EMR) system and picture archiving and communication system (PACS). The inclusion criteria were as follows: (a) patients who underwent EVAR; and (b) patients who received follow-up CT scans (unenhanced and contrast-enhanced CT scans) at least 1 month after EVAR. After querying clinical electronic records, retrieving the image examination records, and reviewing the CT images, we excluded 366, 116, and 28 patients respectively. A total of 216 patients with infrarenal AAA were finally included in this study (Fig. 1). The endovascular grafts used in these patients were bifurcated stent grafts. All enrolled patients had undergone both unenhanced and contrast-enhanced CT examinations (Fig. 2A). Details of patient selection and CT protocols are described in Supplementary Materials.

### Image evaluation

Two vascular imaging radiologists (with 12 and 2 years of experience in the interpretation of vascular CT studies, respectively) independently evaluated the presence or absence of endoleaks based on the enhanced CT images (Fig. 2B). Both reviewers have experience in radiomics analysis. Ambiguous results were discussed together to reach a conclusion by consensus. The results of the consensus reading were used for further analysis.

The reference standard for detecting endoleaks was the presence of persistent blood flow within the aneurysm sac outside the stent on enhanced CT images. Meanwhile, according to previous works of literature, the combination of contrast-enhanced and unenhanced CT can help differentiate small endoleaks from calcified aortic walls, intrathrombus calcifications, or metallic portion of the stent grafts and improve the accuracy of endoleak detection compared with enhanced CT alone [18, 19]. Therefore, in this study, the unenhanced CT scans were also referenced during the endoleak evaluation to help eliminate indeterminate results. According to the image evaluation results, the enrolled patients were divided into two groups: patients with endoleak and patients without endoleak.

### Image segmentation

Three-dimensional AAA was segmented from unenhanced CT scans through a semiautomatic method based on the threshold technique (Fig. 2C). The segmentation range was from the level of the lower renal artery to the bifurcation of the abdominal aorta [20]. The segmentation target was the aneurysm sac outside the stent [16]. The CT attenuation range used for automatic segmentation of the aneurysm sac was defined from − 50 Hounsfield unit (HU) to 150 HU [21–23]. The upper threshold of 150 HU was used to remove tissues with CT values higher than the aneurysms, such as bone, calcified plaque, and metallic portion of stent grafts. The lower threshold of − 50 HU was defined to remove tissues with lower CT values, such as air and adipose tissue. The automatic segmentation results were then manually corrected by two radiologists to eliminate interfering structures caused by missegmentation. The above process was realized through MATLAB (version R2020b, MathWorks) [16, 23, 24].

### Feature extraction

Radiomic features extracted from the aneurysm region included histogram features, texture features, and filtered image features (Fig. 2D). Histogram features, which are also called first-order features, include 20 statistics describing the distribution of CT attenuation within the interested region [11]. Texture features include 75 statistics quantifying the relationships between voxels and their surroundings of both distance and intensity [25]. Filtered images contain 20 different images which are generated through various filters [25]. Each of the filtered images can provide 93 features. In total, 1955 (20 + 75 + [20 × 93]) features were extracted from the unenhanced CT through the Python (version 3.7, Python Software Foundation) program. A detailed description of radiomic features can be found in Supplementary Materials.

### Statistical analysis

Interreader agreements for endoleak evaluation were evaluated using Cohen’s kappa coefficient. The reproducibility of manual correction of the aneurysm segmentation results was assessed by the Dice coefficient [26].

To explore the differences in unenhanced CT features between patients with and without endoleak, we analyzed the 20 histogram features of the two groups (Fig. 2E, experiment 1). Compared with texture features and filtered image features, histogram features have better stability (features that remain the same when imaged multiple times using different equipment, software, or acquisition settings) and interpretability (features defined by basic and complete definitions and formulas) [27]. The Shapiro–Wilk test was used to assess the normality of distribution. Features of normal distribution were analyzed using Student’s *t* test, otherwise using the Mann–Whitney *U* test. *p* values greater than 0.05 on the Shapiro–Wilk test represent a normal distribution. For other tests, *p* values less than 0.05 were considered to indicate significant differences. Statistical analysis was completed by SPSS (version 26.0, International Business Machines Corporation).

### Machine learning

To investigate whether unenhanced CT radiomics can help detect endoleaks, we developed twelve common ML models based on all the extracted features (Fig. 2E, experiment 2). The ML models included adaptive boosting (AB), bagging (BAG), decision tree (DT), gradient boosting (GB), Gaussian Naive Bayes (GNB), *k*-nearest neighbor (KNN), linear discriminant analysis (LDA), logistic regression (LR), neural network (NN), quadratic discriminant analysis (QDA), random forest (RF), and support vector machine (SVM) [28–30].

The process of ML was as follows (Supplementary Materials): **a** divide patients into a training set (80%) and an internal test set (20%) according to the chronological order of CT examination. **b** Standardize the data of the two sets based on *Z*-score normalization. **c** Univariable analysis was performed on the training set data to retain features with *p* < 0.05. **d** Least absolute shrinkage and selection operator (LASSO) regression was used to realize feature dimensionality reduction [31]. **e** According to the final selected features, the twelve ML models were constructed on the training set data [32]. **f** The ML models were tested on the internal test set. All the above processes were implemented by Python programming.

## Results

### Reproducibility

Agreement between the two radiologists was excellent for endoleak evaluation (Cohen’s kappa coefficient, 0.91; 95% CI: 0.85, 0.97; *p* < 0.001) and aneurysm segmentation (Dice coefficient, 0.94 ± 0.05). After comparing and analyzing the evaluation results of the two radiologists, we found that all the incorrect results appeared in the analysis of the junior reviewer. For example, the junior radiologist correctly evaluated the presence or absence of endoleaks in 208 patients and incorrectly evaluated 8 patients, and the senior reviewer correctly analyzed the situation of endoleaks in all 216 patients of the study cohort.

### Differences in unenhanced CT images between patients with and without endoleak

A total of 216 patients (69 ± 8 years; 191 men) were included in this study (Fig. 1). Among them, 64 patients (30%, 64/216; 69 ± 10 years; 51 men) had AAAs containing endoleaks (20 type I and 44 type II), and the other 152 patients (69 ± 7; 140 men) had no endoleaks.

Table 1 presents statistics of the 20 histogram features of patients with or without endoleak. There were 10 features that showed significant differences between the two groups (with endoleak vs. without endoleak): mean (41.7 vs. 33.6, *p* < 0.001), minimum (− 33.9 vs. − 39.1, *p* < 0.001), maximum (156 vs. 149, *p* = 0.004), median (41 vs. 33, *p* < 0.001), 10th percentile (16 vs. 11, *p* < 0.001), 90th percentile (65 vs. 58, *p* < 0.001), mode (41 vs. 32, *p* < 0.001), root mean squared (47.0 vs. 39.5, *p* < 0.001), energy (2.9 × 10^7^ vs. 1.8 × 10^7^, *p* = 0.03), and coefficient of variation (51.5 vs. 58.8, *p* < 0.001). The first nine features, which measure the intensity of CT attenuation values from different aspects, were highly consistent with each other, and all showed higher values in the endoleak group. A lower value of the coefficient of variation represents a small dispersion of frequency distribution, which indicates that CT values of the endoleak group were more concentrated than those of patients without endoleak. For the other 10 histogram features, we found no evidence of a difference between the two groups, and detailed results can be found in Table 1.

**Table 1:** Statistical analysis of histogram features extracted from unenhanced CT slices

Histogram features	All (*n* = 216)	With endoleak (*n* = 64)	Without endoleak (*n* = 152)	*p* value
10th percentile	12 (7, 17)	16 (11, 21)	11 (5, 15)	< 0.001*
90th percentile	59 (54, 65)	65 (60, 70)	58 (52, 63)	< 0.001*
CV	56.2 (48.9, 66.7)	51.5 (45.1, 58.8)	58.8 (51.1, 70.2)	< 0.001*
Energy (× 10^7^)	2.3 (1.2, 4.4)	2.9 (1.3, 5.2)	1.8 (1.1, 4.2)	0.03*
Entropy	1.8 (1.6, 1.9)	1.8 (1.6, 2.0)	1.8 (1.6, 1.9)	0.47
IQR	23 (20, 26)	24 (20, 27)	23 (20, 26)	0.40
Kurtosis	4.7 (4.3, 5.7)	4.7 (4.3, 5.6)	4.7 (4.2, 5.7)	0.79
Maximum	151 (139, 160)	156 (146, 164)	149 (135, 159)	0.004*
Mean	35.8 (30.9, 40.7)	41.7 (37.4, 44.9)	33.6 (29.8, 38.3)	< 0.001*
MAD	15.0 (13.3, 17.0)	15.3 (13.3, 17.3)	14.9 (13.3, 16.8)	0.44
Median	35 (30, 40)	41 (38, 44)	33 (29, 38)	< 0.001*
Minimum	− 37.6 ± 10.3	− 33.9 ± 10.7	− 39.1 ± 9.7	< 0.001*
Mode	35 (29, 40)	41 (36, 44)	32 (28, 37)	< 0.001*
Range	188 (176, 199)	189 (176, 199)	187 (176, 199)	0.46
RMAD	9.8 (8.7, 11.2)	9.9 (8.7, 11.5)	9.7 (8.7, 11.0)	0.40
RMS	40.9 (36.9, 46.6)	47.0 (42.5, 49.8)	39.5 (36.2, 43.4)	< 0.001*
Skewness	0.4 (0.2, 0.6)	0.4 (0.2, 0.6)	0.4 (0.2, 0.7)	0.36
SD	20.2 (17.9, 22.7)	20.6 (17.6, 23.1)	19.8 (17.9, 22.5)	0.45
Uniformity	0.4 ± 0.1	0.4 ± 0.1	0.4 ± 0.1	0.42
Variance	407 (321, 515)	423 (311, 533)	393 (321, 507)	0.45

Abbreviations: *CV*, coefficient of variation; *IQR*, interquartile range; *MAD*, mean absolute deviation; *RMAD*, robust mean absolute deviation; *RMS*, root mean squared; *SD*, standard deviation

Unless otherwise indicated, data are mean ± standard deviation or median (interquartile range). *p values less than 0.05

**Fig. 3:**
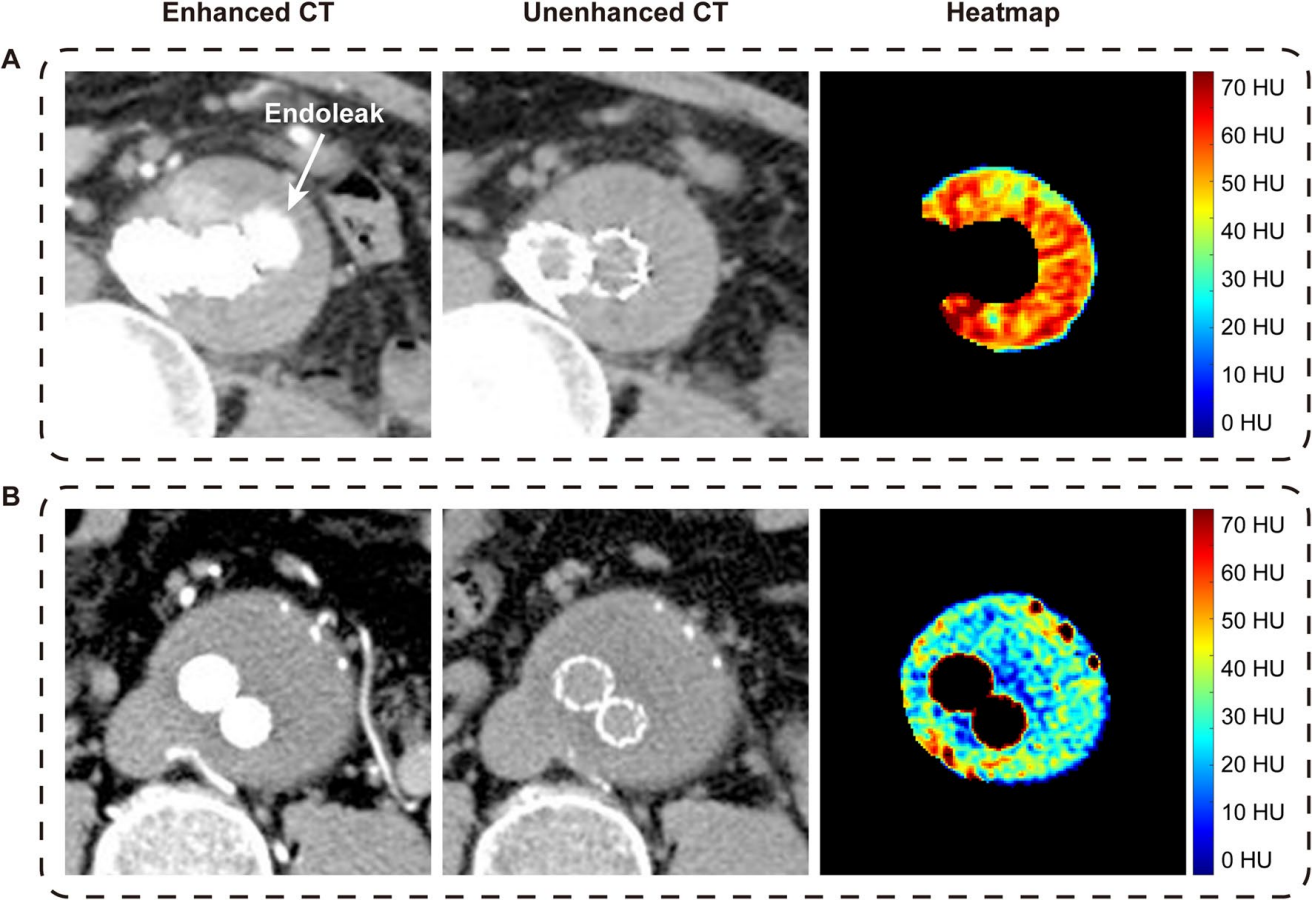
Examples of CT attenuation of patients with or without endoleak. **A** A 69-year-old man with endoleak after endovascular repair. The first image was the arterial phase enhanced CT on the maximal axial plane of the abdominal aortic aneurysm. The second image was the unenhanced CT slice at the same level as the first one. The third image was the heatmap of CT attenuation of the aneurysm sac segmented from the second image. The red area of the color bar reflects high CT values and the blue reflects low values. **B** A 64-year-old man in the nonendoleak group. No endoleak was observed in the follow-up CT scans after endovascular repair. Images of **B** represent the same meaning as **A**. HU, Hounsfield unit

Figure 3 shows two examples of CT values of patients with or without endoleak. Comparing the two heatmaps of AAA, the aneurysm with endoleak is shown as red regions with higher CT values, and the aneurysm without endoleak is shown as blue regions with lower values. In Fig. 3, we can visually see that CT attenuation values of unenhanced CT in the endoleak group are significantly higher than those in the without endoleak group.

### Endoleak detection with radiomic features from unenhanced CT images

Based on the examination time (2014.7.22–2021.9.14) of follow-up CT images, the patients were divided into a training set (2014.7.22–2019.10.25) and an internal test set (2019.10.26–2021.9.14). The training set contained 173 patients (80%, 173/216) with 52 (30%, 52/173) endoleaks (17 type I and 35 type II). The test set had 43 patients (20%, 43/216) with 12 (28%, 12/43) endoleaks (3 type I and 9 type II). The distribution of endoleaks was balanced in the two datasets and was almost the same as the endoleak proportion in the entire study cohort (30%, 64/216).

**Fig. 4:**
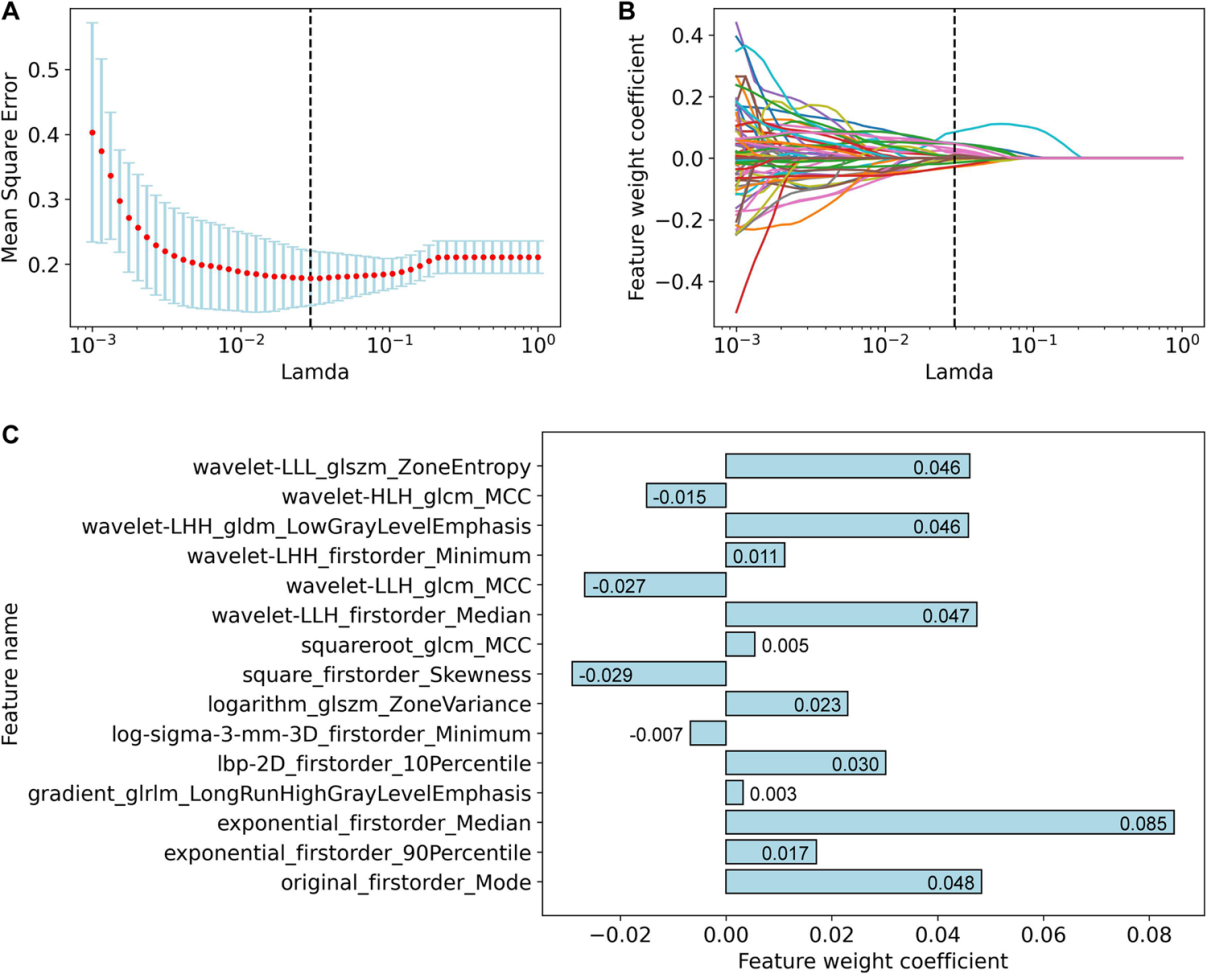
Feature dimensionality reduction. **A** The trend graph of mean square error with *λ* (Lamda) during cross-validation. *λ* is an important parameter of LASSO regression that is usually adjusted by cross-validation to find the optimal value. The red dots represent the average values of the mean square error. The blue error bars represent the standard deviation. The black dotted line indicates the best value of *λ*. **B** The convergence graph of weight coefficients of the 206 radiomic features, which were selected through univariable analysis. As shown in **A** and **B**, the mean square error is minimized (0.18 ± 0.04) at *λ* = 0.029 (the black dotted line), where 15 representative radiomic features were finally identified (weight coefficient ≠ 0). **C** Feature names and weight coefficients of the 15 final selected features. GLCM, gray-level co-occurrence matrix, GLDM, gray-level dependence matrix, GLRLM, gray-level run-length matrix, GLSZM, gray-level size zone matrix

We performed a univariable analysis on the 1955 CT features of the training set and retained 206 features with *p* < 0.05 (Supplementary Table S1), including ten histogram features, four texture features, and 192 filtered image features. The ten histogram features were identical to the statistics with *p* < 0.05 in experiment 1. Figure 4 shows the results of feature dimensionality reduction through LASSO regression. Finally, 15 representative radiomic features were identified (Fig. 4C).

**Fig. 5:**
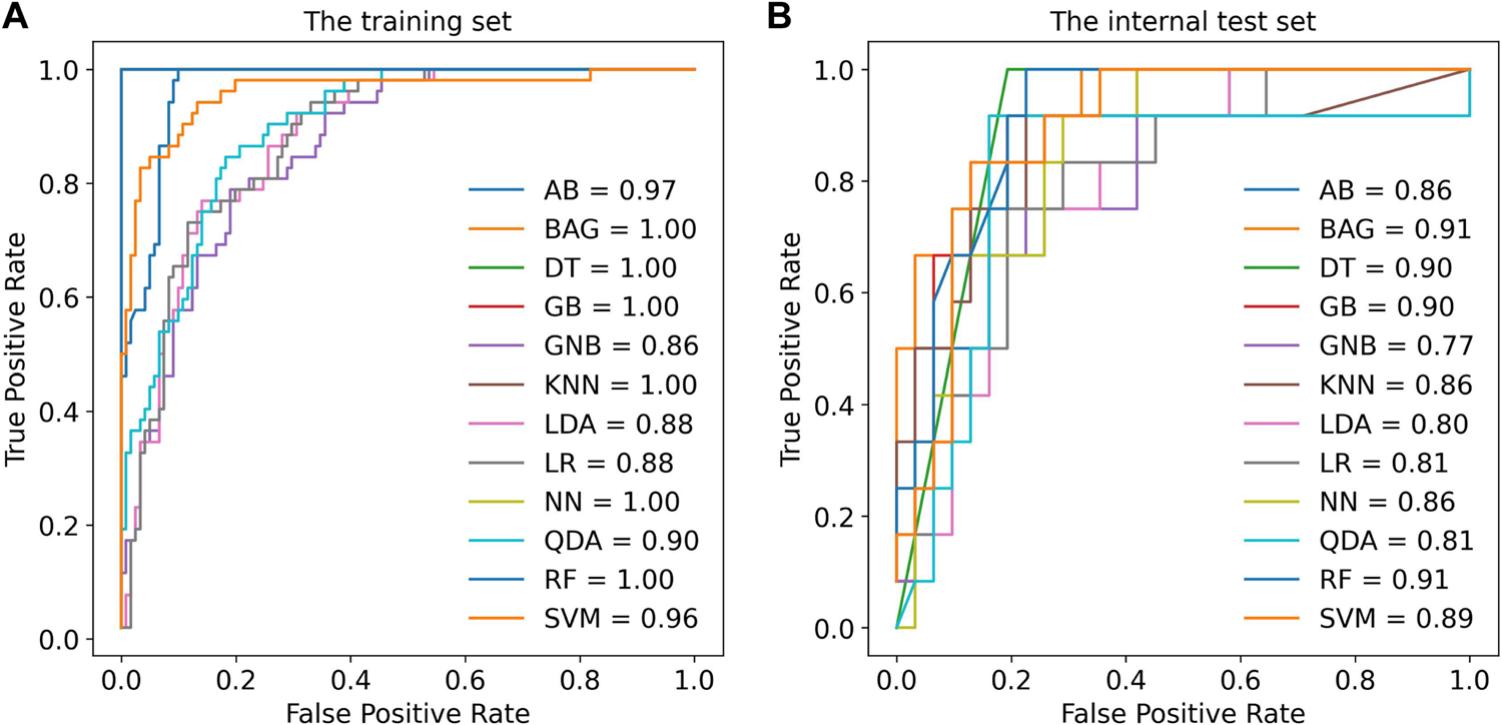
Performance of the machine learning models for endoleak detection. **A** Receiver operating characteristic (ROC) curves of the training set. **B** ROC curves of the internal test set. AB, adaptive boosting, BAG, bagging, DT, decision tree, GB, gradient boosting, GNB, Gaussian Naive Bayes, KNN, *k*-nearest neighbors, LDA, linear discriminant analysis, LR, logistic regression, NN, neural network, QDA, quadratic discriminant analysis, RF, random forest, SVM, support vector machine

Based on the 15 selected features, we developed twelve common ML models for endoleak detection (Supplementary Table S2). Figure 5 presents the receiver operating characteristic (ROC) curves of the training set and the internal test set. The average area under the curve (AUC) of the models on the training set was 0.95 ± 0.05 with a classification accuracy of 93% ± 8, and the AUC of the test set was 0.86 ± 0.05 with an accuracy of 81% ± 4. Table 2 shows the detailed detection performance of the ML models on the internal test set. Four of the twelve models had an AUC greater than 0.90: BAG (AUC = 0.91), RF (AUC = 0.91), DT (AUC = 0.90), and GB (AUC = 0.90). The other seven models had an AUC greater than 0.80: SVM (AUC = 0.89), AB (AUC = 0.86), KNN (AUC = 0.86), NN (AUC = 0.86), LR (AUC = 0.81), QDA (AUC = 0.81), and LDA (AUC = 0.80). GNB obtained the lowest AUC of 0.77.

**Table 2:** Performance of the radiomic models for endoleak detection on the internal test set

Model	AUC (95% CI)	*p* value	Accuracy	Sensitivity	Specificity	Cut-point
AB	0.86 (0.74, 0.98)	< 0.001	79 (34/43)	92 (11/12)	74 (23/31)	0.48
BAG	0.91 (0.83, 1.00)	< 0.001	77 (33/43)	100 (12/12)	68 (21/31)	0.30
DT	0.90 (0.81, 0.99)	< 0.001	86 (37/43)	100 (12/12)	81 (25/31)	1.00
GB	0.90 (0.80, 0.99)	< 0.001	84 (36/43)	92 (11/12)	81 (25/31)	0.23
GNB	0.77 (0.60, 0.94)	0.006	77 (33/43)	75 (9/12)	77 (24/31)	0.11
KNN	0.86 (0.71, 1.00)	< 0.001	81 (35/43)	92 (11/12)	77 (24/31)	0.41
LDA	0.80 (0.66, 0.93)	0.003	79 (34/43)	67 (8/12)	84 (26/31)	0.38
LR	0.81 (0.67, 0.94)	0.002	79 (34/43)	75 (9/12)	81 (25/31)	0.50
NN	0.86 (0.75, 0.97)	< 0.001	77 (33/43)	92 (11/12)	71 (22/31)	0.32
QDA	0.81 (0.64, 0.98)	0.002	86 (37/43)	92 (11/12)	84 (26/31)	0.31
RF	0.91 (0.83, 0.99)	< 0.001	84 (36/43)	100 (12/12)	77 (24/31)	0.34
SVM	0.89 (0.79, 0.99)	< 0.001	86 (37/43)	83 (10/12)	87 (27/31)	0.41

Abbreviations: *AUC*, area under the curve; *CI*, confidence interval; *AB*, adaptive boosting; *BAG*, bagging; *DT*, decision tree; *GB*, gradient boosting; *GNB*, Gaussian Naive Bayes; *KNN*, *k*-nearest neighbors; *LDA*, linear discriminant analysis; *LR*, logistic regression; *NN*, neural network; *QDA*, quadratic discriminant analysis; *RF*, random forest; *SVM*, support vector machine

The selection criterion of the cut-points: the highest value of sensitivity + specificity-1

**Fig. 6:**
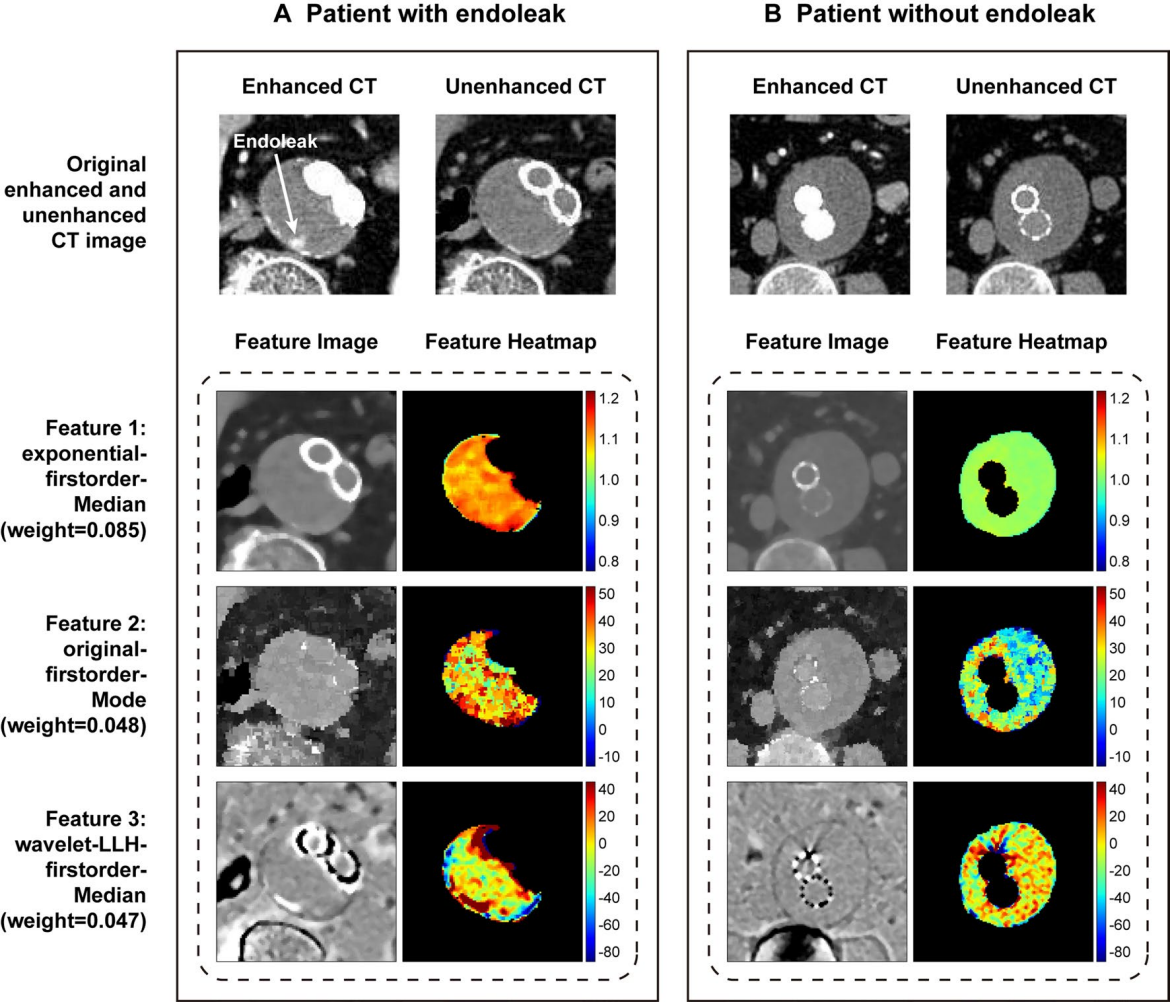
Visualization of unenhanced CT radiomic features. **A** An 89-year-old woman with endoleak after endovascular repair in the test set. The first image in the top row was the arterial phase enhanced CT slice on the maximal axial plane of the abdominal aortic aneurysm. The second image in the top row was the unenhanced CT slice at the same level as the first one. The other images below were feature images and heatmaps of the three radiomic features with the highest weight coefficients extracted from unenhanced CT images. The red area of the color bar reflects high values and the blue reflects low values. **B** A 67-year-old man without endoleak in the test set. Images of **B** represent the same meaning as **A**

Figure 6 shows the visualization of the first three features with the highest coefficients. Although the aneurysm in Fig. 6A had a small endoleak, which is not conspicuous in the contrast-enhanced CT image, the heatmaps of unenhanced CT features are still measurably different from the aneurysm without endoleak in Fig. 6B.

### Clinical management strategies

The common selection criterion of cut-points is the highest value of the Youden index (sensitivity + specificity-1), which assumes that sensitivity and specificity are of equal value in clinical management. The performance of the twelve detection models under this criterion is shown in Table 2. The average sensitivity was 88% ± 10, and 9 of them exceeded 80% (BAG = 100%, DT = 100%, RF = 100%, AB = 92%, GB = 92%, KNN = 92%, NN = 92%, QDA = 92%, and SVM = 83%). The average specificity was 78% ± 5, and 6 of them exceeded 80% (SVM = 87%, LDA = 84%, QDA = 84%, DT = 81%, GB = 81%, LR = 81%).

To consider clinical strategies under various circumstances, we evaluated the detection capability of the radiomic models on different selection criteria (Table 3). When fixing the sensitivity to > 90% to select the cut-points, the models exhibited high sensitivity that may be appropriate for endoleak screening during follow-up. Under this criterion, the average sensitivity of the models was 92% ± 2, and the specificity was 72% ± 10. When fixing the specificity to > 90%, the models showed high specificity (91% ± 3) with an average sensitivity of 51% ± 20. Details of the evaluation results can be found in Table 3.

**Table 3:** Sensitivity and specificity of the detection models under different clinical strategies

Model	Fixed sensitivity > 90			Fixed specificity > 90		
	Sensitivity	Specificity	Cut-point^†^	Sensitivity	Specificity	Cut-point^‡^
AB	92 (11/12)	74 (23/31)	0.47	50 (6/12)	90 (28/31)	0.49
BAG	92 (11/12)	74 (23/31)	0.34	67 (8/12)	90 (28/31)	0.54
DT	100 (12/12)	81 (25/31)	0.50	00 (0/12)	100 (31/31)	2.00
GB	92 (11/12)	81 (25/31)	0.21	67 (8/12)	90 (28/31)	0.50
GNB	92 (11/12)	58 (18/31)	0.01	42 (5/12)	90 (28/31)	0.91
KNN	92 (11/12)	77 (24/31)	0.40	58 (7/12)	90 (28/31)	0.61
LDA	92 (11/12)	55 (17/31)	0.12	42 (5/12)	90 (28/31)	0.57
LR	92 (11/12)	55 (17/31)	0.43	42 (5/12)	90 (28/31)	0.54
NN	92 (11/12)	71 (22/31)	0.20	67 (8/12)	90 (28/31)	0.92
QDA	92 (11/12)	84 (26/31)	0.25	33 (4/12)	90 (28/31)	0.96
RF	92 (11/12)	81 (25/31)	0.39	67 (8/12)	90 (28/31)	0.53
SVM	92 (11/12)	74 (23/31)	0.31	75 (9/12)	90 (28/31)	0.44

Abbreviations: *AB*, adaptive boosting; *BAG*, bagging; *DT*, decision tree; *GB*, gradient boosting; *GNB*, Gaussian Naive Bayes; *KNN*, *k*-nearest neighbors; *LDA*, linear discriminant analysis; *LR*, logistic regression; *NN*, neural network; *QDA*, quadratic discriminant analysis; *RF*, random forest; *SVM*, support vector machine

^†^The selection criterion of the cut-point: fixed sensitivity > 90%. ^‡^The selection criterion of the cut-point: fixed specificity > 90%

## Discussion

In this study, we collected CT imaging data of patients with infrarenal AAA after EVAR to investigate the feasibility of endoleak detection using unenhanced CT images. Through extracting and analyzing the radiomic features, we explored the differences in unenhanced CT images between patients with and without endoleak and found that aneurysms of the endoleak group showed an obviously higher CT value with smaller dispersion than the nonendoleak group. Furthermore, twelve common ML models were constructed using the unenhanced radiomic features to validate the performance of endoleak detection, reaching an average AUC of 0.86 ± 0.05 in the internal test set.

Since the CT attenuation of blood is usually higher than those of soft tissues, it is reasonable that endoleaks caused elevated unenhanced CT values in the aneurysm sac. This result verifies our conjecture in the “Introduction” that the differences in aneurysm composition caused by endoleak are indeed reflected on unenhanced CT images and can be “observed” through the method of radiomics. Meanwhile, the evaluation results of the twelve ML models indicate that unenhanced CT features have the potential to detect endoleaks and are applicable to most common ML methods. At present, there are few studies on endoleak detection by using unenhanced CT scans, and our results can provide support for the feasibility of this research direction.

The radiomic models yielded a relatively high sensitivity of 88% ± 10 with 78% ± 5 specificity. When fixing the sensitivity to > 90% (92% ± 2) [33, 34], the average specificity was 72% ± 10. High sensitivity also resulted in a low missed diagnosis rate. This indicates that the unenhanced CT radiomics have the potential to provide high-sensitivity outcomes and can be used to assist in postoperative follow-up. For example, we can establish the following clinical strategy of postoperative monitoring: after EVAR, patients are first scanned with unenhanced CT during follow-up; if the output of the radiomics model is positive, a further enhanced CT examination is performed to confirm the presence of endoleak; if the output is negative, the patient is no longer scanned with contrast-enhanced CT, and follow-up continues at regular intervals. The above procedure can reduce the injection of contrast agents in patients and avoid adverse reactions such as nephrotoxicity as much as possible.

For the high sensitivity of the detection models, we analyzed the possible reasons as follows: (a) nine of the ten histogram features with significant differences in experiment 1 showed higher values in the endoleak group; (b) eleven of the fifteen features finally screened through LASSO had positive weight coefficients, which included the five features with the highest weights. From the above two results, we can see that the majority of unenhanced CT features associated with the existence of endoleaks are “risk factors” rather than “protective factors.” And most of the risk factors are finally retained in the ML models with a high coefficient. Therefore, the unenhanced CT radiomic models may tend to be sensitive to the existence of endoleaks, which leads to a high-sensitivity outcome.

At present, there are many studies involving the detection of endoleaks after EVAR. As part of these works of research, the detection performance of various contrast-enhanced CT, such as spectral photon-counting CT [35], dual-energy low-keV or single-energy low-kV CT [4], and sparse sampling CT [36], has been investigated. Other researchers have explored endoleak detection with contrast-enhanced ultrasound (CEUS) or magnetic resonance angiography (MRA).

In a comparative retrospective study, CEUS reported a sensitivity of 97.4% in endoleak detection using CT angiography (CTA) as a reference standard [37]. Compared with CTA, the contrast medium used in CEUS is not nephrotoxic and had a low risk for patients [38]. However, a major drawback of CEUS is operator dependence, which reduces the availability of CEUS [39]. Meanwhile, due to the poor visibility caused by increased fatty tissue, the missed endoleaks on CEUS are overrepresented in patients with high BMIs [40].

In another study on MRA, sensitivity, specificity, and accuracy for endoleak detection were 77.3%, 91.7%, and 84.8%, respectively [41]. MRA can recognize the presence of endoleaks in aneurysms, but contraindications, such as claustrophobia, some other types of metal implants, and the presence of implanted pacemakers, limited the use of MRA to some extent [41]. Therefore, contrast-enhanced CT remains extensively used in clinical practice owing to its excellent reproducibility and spatial and/or contrast resolution. However, few studies have verified the possibility of endoleak detection with unenhanced CT.

The significance of this study is as follows: (a) provide an alternative method of endoleak detection in patients who have adverse reactions to contrast media. (b) Provide an objective and automatic method to detect endoleaks, which avoids the bias introduced by subjective reading. (c) Further exploit the value of unenhanced CT examinations in the clinical management and surveillance of postoperative AAA.

There are several limitations to our work. (a) As this is a retrospective study, we still need to conduct large-sample and prospective studies to further evaluate and verify this method. (b) We were limited by the development of image segmentation technology; three-dimensional aneurysm segmentation still needs manual correction and cannot be achieved completely through automatic procedures at present. (c) The ML models were not evaluated on the external test set, so the applicability of this method still needs further verification. (d) We only explored the detection of the presence of endoleaks with unenhanced CT but did not further study the diagnosis of a specific endoleak type. The practical application of unenhanced CT in the postoperative follow-up of patients with AAA still needs more exploration and investigation. (e) In the actual clinical practice, endoleaks that do not contribute to the AAA increase or endoleak intervention are of little clinical importance. Therefore, the lack of analysis of whether the radiomic model could predict clinically significant endoleaks is also a drawback of our study.

In conclusion, there are differences in unenhanced CT images of abdominal aortic aneurysm between patients with and without endoleak. Machine learning models constructed with unenhanced radiomic features can help detect the presence of endoleaks with high sensitivity. In the future, we will further explore the feasibility of diagnosis of endoleak type (aggressive or benign) using unenhanced CT and fully exploit the potential value of CT examinations in postoperative follow-up of patients with aneurysms.

### Supplementary Information

Below is the link to the electronic supplementary material.

Supplementary file1 (PDF 346 kb)

## Abbreviations

AAA: Abdominal aortic aneurysm
AB: Adaptive boosting
BAG: Bagging
DT: Decision tree
EVAR: Endovascular aneurysm repair
GB: Gradient boosting
GNB: Gaussian Naive Bayes
KNN: *k*-Nearest neighbor
LASSO: Least absolute shrinkage and selection operator
LDA: Linear discriminant analysis
LR: Logistic regression
ML: Machine learning
NN: Neural network
QDA: Quadratic discriminant analysis
RF: Random forest
SVM: Support vector machine

## Declarations

### Guarantor

The scientific guarantor of this publication is Zhiwei Wang.

### Statistics and Biometry

No complex statistical methods were necessary for this paper.

### Methodology

• Retrospective

• Case–control study

• Performed at one institution
