# Supplementary material for: Unenhanced computed tomography radiomics help detect endoleaks after endovascular repair of abdominal aortic aneurysm
Source: Eur Radiol. 2023 Sep 2;34(3):1647–58. doi: 10.1007/s00330-023-10000-y (PMC10873228; doi:10.1007/s00330-023-10000-y)
Supplement: Supplementary file 1 — Supplementary file1 (PDF 346 kb) [file 330_2023_10000_MOESM1_ESM.pdf]

# Unenhanced computed tomography radiomics help detect endoleaks after endovascular repair of abdominal aortic aneurysm

**Electronic Supplementary Material (ESM)**

*Eur Radiol (2023) Hu G, Ding N, Wang Z, Jin Z*

## Patient selection

We initially collected 726 patients with abdominal aortic aneurysm (AAA) who visited our hospital from July 2014 to September 2021. The inclusion criteria were as follows: (a) patients who underwent endovascular aneurysm repair (EVAR); and (b) patients who received follow-up CT scans (unenhanced and contrast-enhanced CT scans) at least one month after EVAR. By querying electronic medical records, we excluded (a) patients whose surgical records were unavailable (n=335); (b) patients who underwent open aneurysm repair (n=14); (c) patients with thoracoabdominal aortic aneurysm (n=11); and (d) patients with isolated iliac aneurysm (n=6). After retrieving the image examination records of the patients, we made the following exclusions: (e) patients who did not receive follow-up CT scans (n=84); (f) patients with insufficient follow-up duration (< one month, n=9); (g) patients with no contrast-enhanced CT scans (n=21); and (h) patients with no unenhanced CT scans (n=2). According to the image quality, we excluded (i) aneurysm sacs that were too small for feature extraction (n=14); (j) images with severe motion artifacts (n=7); and (k) patients with ruptured AAA (n=7). Finally, a total of 216 patients with infrarenal AAA were included in this study.

## CT protocol

All enrolled patients had undergone unenhanced and enhanced CT examinations, which were mainly performed using a dual-source CT scanner

(SOMATOM Definition Flash, Siemens Healthcare). The imaging protocol was biphasic, with a head-first supine position and a scan length from the diaphragm level to the pubic symphysis level. After scanning unenhanced CT images, the contrast medium (370 mgI/mL, Iopromide, Ultravist, Bayer Healthcare) was injected through the median right elbow at a rate of 4 ml/s. A bolus tracking technique (trigger threshold, 100 HU; trigger level, the abdominal aorta) was used to trigger the scanning of enhanced CT examinations. All acquisitions were reconstructed with thicknesses of 1 mm (for image evaluation) and 5 mm (for image segmentation) based on the Digital Imaging and Communications in Medicine (DICOM) standard, and the size of each CT slice was 512×512 pixels.

**Radiomic features**

a) ***Histogram features***, which are also called first-order features, describe the intensity and distribution of CT attenuation values within the interested region (the aneurysm sac outside the stent) defined by the mask through commonly used and basic metrics, including the following 20 statistics.

- $10^{th}$ Percentile: the $10^{th}$ percentile CT attenuation within the aneurysm region of interest (ROI).
- $90^{th}$ Percentile: the $90^{th}$ percentile CT attenuation within the ROI.
- Coefficient of variation (CV): CV also known as relative standard deviation, is a standardized measure of the dispersion of a probability distribution or frequency distribution of CT attenuation.

- Energy: energy is a measure of the magnitude of voxel values in a CT image. A larger value implies a greater sum of the squares of these values.
- Entropy: entropy specifies the uncertainty/randomness in the CT attenuation. It measures the average amount of information required to encode the CT values.
- Interquartile Range: the CT values between the 25$^{th}$ and 75$^{th}$ percentile.
- Kurtosis: kurtosis is a measure of the peakedness of the distribution of CT attenuation in the ROI. A higher kurtosis implies that the mass of the distribution is concentrated towards the tail(s) rather than towards the mean. A lower kurtosis implies the reverse: that the mass of the distribution is concentrated towards a spike near the Mean value.
- Maximum: the maximum CT attenuation within the ROI.
- Mean: the average CT value within the ROI.
- Mean absolute deviation (MAD): MAD is the mean distance of all CT values from the mean value of the CT image array.
- Median: the median CT value within the ROI.
- Minimum: the minimum CT attenuation within the ROI.
- Mode: the mode CT value within the ROI.
- Range: the range of CT values in the ROI.
- Robust mean absolute deviation (RMAD): RMAD is the mean distance of all CT values from the Mean Value calculated on the subset of image

array with CT values in between, or equal to the 10$^{th}$ and 90$^{th}$ percentile.

- Root mean squared (RMS): RMS is the square-root of the mean of all the squared CT attenuation. It is another measure of the magnitude of the image values.
- Skewness: skewness measures the asymmetry of the distribution of CT attenuation about the mean value. Depending on where the tail is elongated and the mass of the distribution is concentrated, this value can be positive or negative.
- Standard Deviation (SD): SD measures the amount of variation or dispersion from the mean value.
- Uniformity: uniformity is a measure of the sum of the squares of each CT attenuation. This is a measure of the homogeneity of the image array, where a greater uniformity implies a greater homogeneity or a smaller range of discrete CT values.
- Variance: Variance is the mean of the squared distances of each CT attenuation from the mean value. This is a measure of the spread of the distribution about the mean.

b) ***Texture features*** quantify the relationship between voxels and their surroundings of both distance and intensity, containing 75 statistics in the following 5 categories.

- Gray level co-occurrence matrix (GLCM) features (24 features): GLCM describes the second-order joint probability function of an image region

constrained by the matrix that computes how often pairs of pixels with a specific value and offset occur in the image.

- Gray level dependence matrix (GLDM) features (14 features): GLDM quantifies gray level dependencies in an image. A gray level dependency is defined as the number of connected voxels within distance δ that are dependent on the center voxel.
- Gray level run length matrix (GLRLM) features (16 features): GLRLM quantifies gray level runs, which are defined as the length in number of pixels, of consecutive pixels that have the same CT value.
- Gray level size zone matrix (GLSZM) features (16 features): GLSZM quantifies CT attenuation zones in an image. A CT attenuation zone is defined as the number of connected voxels that share the same gray level intensity. A voxel is considered connected if the distance is 1 according to the infinity norm.
- Neighboring gray tone difference matrix (NGTDM) features (5 features): NGTDM quantifies the difference between a CT value and the average CT value of its neighbors within distance δ.

c) ***Filtered images*** are generated after the original image is processed by different filters, including the following 20 images in this research. Each of the images provides 93 radiomic features.

- Exponential image: computes the exponential of the original image.
- Gradient image: compute and return the gradient magnitude in the CT

image considering the image spacing.

- Local binary pattern (LBP) 2D image: compute and return the LBP of the original CT image in 2D.
- LBP 3D images (m1, m2, k): compute and return the LBP of the original CT image in 3D using spherical harmonics. Yields LBP filtered image for each level (LBP-3D-m1 and LBP-3D-m2) and yields the kurtosis image (LBP-3D-k).
- Logarithm image: computes the logarithm of the absolute CT value of the original image + 1.
- Laplacian of Gaussian (LoG) images (sigma = 1, 2, 3): applies a LoG filter to the original CT image and yields a derived image for each sigma value specified.
- Square image: computes the square of the CT attenuation values.
- Square root image: computes the square root of the absolute value of CT attenuation.
- Wavelet images (LLL, LLH, LHL, LHH, HLL, HLH, HHL, and HHH): applies wavelet filter to the original CT image and yields the decompositions and the approximation. The wavelet filter takes all possible combinations of low-pass (L) or high-pass (H) filters.

**Machine learning**

a) ***Dataset division:*** The patients were sorted according to the chronological

order of CT examination. The first 80% of patients were divided into the training set, and the last 20% were divided into the internal test set. The advantage of dividing datasets in chronological order is that the test set has the characteristics of “prospective” to a certain extent, which increases the reliability of model evaluation.

b) ***Data standardization:*** Standardize the data of the training set and the test set based on the Z-score. This method normalizes the data according to the mean and standard deviation of the original data. The processed data conforms to the standard normal distribution, the mean value is 0 and the standard deviation is 1. Through Z-score standardization, the data of different orders of magnitude are uniformly transformed into the same one, which is measured by the calculated Z-score value to ensure comparability between the data.

c) ***Feature selection:*** Perform univariable analysis on the training set data to retain features with $p<.05$. The Shapiro-Wilk test was first used to assess the normality of distribution. Variables of normal distribution were analyzed using Student’s t test, otherwise using Mann-Whitney U test. P values more than .05 on Shapiro-Wilk test represent normal distribution. For other tests, p values less than .05 were considered to indicate significant differences.

d) ***Feature dimensionality reduction:*** Using least absolute shrinkage and selection operator (LASSO) regression based on 10-fold cross-validation to realize feature dimensionality based on the features selected through the

previous step. LASSO regression is a shrinkage algorithm that allows active selection from a set of variables with a large amount of data and potential multicollinearity to produce more relevant and interpretable features. The important parameter λ with the lowest mean square error is selected by 10-fold cross-validation. In 10-fold cross-validation, the original training set is randomly partitioned into 10 equal-sized subsamples. Of the 10 subsamples, a single subsample is retained as the validation data for testing the model, and the remaining 9 subsamples are used as new training data. The cross-validation process is then repeated 10 times, with each of the 10 subsamples used exactly once as the validation data. The 10 results can then be averaged to produce a single estimation.

e) ***Machine learning (ML):*** According to the final selected radiomic features, twelve common ML models were constructed on the training set. The hyperparameters were tuned using GridSearchCV to stabilize and optimize the model performance. GridSearchCV is a method that encapsulates grid search and cross-validation, which can automatically adjust parameters and return the best combination. Grid search is simply an exhaustive search through a manually specified subset of the hyperparameter space of a learning algorithm. Procedures of cross-validation can refer to the previous section of this material.

f) ***Model evaluation:*** Test and evaluate the performance of ML models on the test set data. Plot receiver operating characteristic (ROC) curves of the

models, and calculate area under the curve (AUC) value with 95% confidence interval, classification threshold (cut-point), sensitivity, specificity, and classification accuracy.

**Supplementary Table S1** Radiomic features selected by univariable analysis (P<0.05)

| No | Radiomic feature name | P value |
|---|---|---|
| 1 | original_firstorder_Mode | 0.000 |
| 2 | original_firstorder_CoefficientVariation | 0.000 |
| 3 | original_firstorder_10Percentile | 0.000 |
| 4 | original_firstorder_90Percentile | 0.000 |
| 5 | original_firstorder_Energy | 0.017 |
| 6 | original_firstorder_Maximum | 0.019 |
| 7 | original_firstorder_Mean | 0.000 |
| 8 | original_firstorder_Median | 0.000 |
| 9 | original_firstorder_Minimum | 0.003 |
| 10 | original_firstorder_RootMeanSquared | 0.000 |
| 11 | original_glcm_Correlation | 0.008 |
| 12 | original_glcm_Imc1 | 0.048 |
| 13 | original_glcm_Imc2 | 0.044 |
| 14 | original_glcm_MCC | 0.039 |
| 15 | exponential_firstorder_10Percentile | 0.000 |
| 16 | exponential_firstorder_90Percentile | 0.000 |
| 17 | exponential_firstorder_Maximum | 0.025 |
| 18 | exponential_firstorder_Mean | 0.000 |
| 19 | exponential_firstorder_Median | 0.000 |
| 20 | exponential_firstorder_Minimum | 0.004 |
| 21 | exponential_firstorder_RootMeanSquared | 0.000 |
| 22 | gradient_firstorder_Kurtosis | 0.024 |
| 23 | gradient_glrlm_LongRunHighGrayLevelEmphasis | 0.022 |
| 24 | gradient_glszm_GrayLevelNonUniformityNormalized | 0.033 |
| 25 | gradient_glszm_ZoneEntropy | 0.032 |
| 26 | lbp-2D_firstorder_10Percentile | 0.034 |
| 27 | log-sigma-1-mm-3D_firstorder_Mean | 0.044 |
| 28 | log-sigma-3-mm-3D_firstorder_Minimum | 0.029 |
| 29 | log-sigma-3-mm-3D_glcm_Autocorrelation | 0.037 |
| 30 | log-sigma-3-mm-3D_glcm_JointAverage | 0.044 |
| 31 | log-sigma-3-mm-3D_glcm_SumAverage | 0.044 |
| 32 | log-sigma-3-mm-3D_gldm_HighGrayLevelEmphasis | 0.041 |
| 33 | log-sigma-3-mm-3D_glrlm_GrayLevelVariance | 0.042 |
| 34 | log-sigma-3-mm-3D_glrlm_HighGrayLevelRunEmphasis | 0.042 |
| 35 | log-sigma-3-mm-3D_glszm_GrayLevelVariance | 0.039 |
| 36 | log-sigma-3-mm-3D_ngtdm_Complexity | 0.029 |
| 37 | logarithm_firstorder_10Percentile | 0.000 |
| 38 | logarithm_firstorder_90Percentile | 0.006 |

| | | |
|---|---|---|
| 39 | logarithm_firstorder_Entropy | 0.000 |
| 40 | logarithm_firstorder_InterquartileRange | 0.000 |
| 41 | logarithm_firstorder_Kurtosis | 0.000 |
| 42 | logarithm_firstorder_MeanAbsoluteDeviation | 0.000 |
| 43 | logarithm_firstorder_Mean | 0.000 |
| 44 | logarithm_firstorder_Median | 0.000 |
| 45 | logarithm_firstorder_Minimum | 0.005 |
| 46 | logarithm_firstorder_RobustMeanAbsoluteDeviation | 0.000 |
| 47 | logarithm_firstorder_RootMeanSquared | 0.000 |
| 48 | logarithm_firstorder_Skewness | 0.000 |
| 49 | logarithm_firstorder_Uniformity | 0.000 |
| 50 | logarithm_firstorder_Variance | 0.000 |
| 51 | logarithm_glcm_ClusterProminence | 0.001 |
| 52 | logarithm_glcm_ClusterShade | 0.000 |
| 53 | logarithm_glcm_ClusterTendency | 0.000 |
| 54 | logarithm_glcm_Contrast | 0.000 |
| 55 | logarithm_glcm_Correlation | 0.044 |
| 56 | logarithm_glcm_DifferenceAverage | 0.000 |
| 57 | logarithm_glcm_DifferenceEntropy | 0.000 |
| 58 | logarithm_glcm_DifferenceVariance | 0.000 |
| 59 | logarithm_glcm_Id | 0.000 |
| 60 | logarithm_glcm_Idm | 0.000 |
| 61 | logarithm_glcm_Idmn | 0.000 |
| 62 | logarithm_glcm_Idn | 0.000 |
| 63 | logarithm_glcm_InverseVariance | 0.000 |
| 64 | logarithm_glcm_JointEnergy | 0.001 |
| 65 | logarithm_glcm_JointEntropy | 0.001 |
| 66 | logarithm_glcm_MaximumProbability | 0.040 |
| 67 | logarithm_glcm_SumEntropy | 0.000 |
| 68 | logarithm_glcm_SumSquares | 0.000 |
| 69 | logarithm_gldm_DependenceNonUniformityNormalized | 0.000 |
| 70 | logarithm_gldm_DependenceVariance | 0.000 |
| 71 | logarithm_gldm_GrayLevelVariance | 0.000 |
| 72 | logarithm_gldm_LargeDependenceEmphasis | 0.000 |
| 73 | logarithm_gldm_LargeDependenceHighGrayLevelEmphasis | 0.001 |
| 74 | logarithm_gldm_LowGrayLevelEmphasis | 0.036 |
| 75 | logarithm_gldm_SmallDependenceEmphasis | 0.000 |
| 76 | logarithm_gldm_SmallDependenceHighGrayLevelEmphasis | 0.028 |
| 77 | logarithm_gldm_SmallDependenceLowGrayLevelEmphasis | 0.030 |
| 78 | logarithm_glrlm_GrayLevelNonUniformityNormalized | 0.000 |
| 79 | logarithm_glrlm_GrayLevelVariance | 0.000 |
| 80 | logarithm_glrlm_LongRunEmphasis | 0.000 |
| 81 | logarithm_glrlm_LongRunLowGrayLevelEmphasis | 0.044 |

| | | |
|---|---|---|
| 82 | logarithm_glrlm_LowGrayLevelRunEmphasis | 0.040 |
| 83 | logarithm_glrlm_RunEntropy | 0.000 |
| 84 | logarithm_glrlm_RunLengthNonUniformityNormalized | 0.000 |
| 85 | logarithm_glrlm_RunPercentage | 0.000 |
| 86 | logarithm_glrlm_RunVariance | 0.000 |
| 87 | logarithm_glrlm_ShortRunEmphasis | 0.000 |
| 88 | logarithm_glrlm_ShortRunLowGrayLevelEmphasis | 0.039 |
| 89 | logarithm_glszm_GrayLevelNonUniformityNormalized | 0.000 |
| 90 | logarithm_glszm_GrayLevelVariance | 0.001 |
| 91 | logarithm_glszm_LargeAreaEmphasis | 0.001 |
| 92 | logarithm_glszm_LargeAreaHighGrayLevelEmphasis | 0.001 |
| 93 | logarithm_glszm_SizeZoneNonUniformityNormalized | 0.000 |
| 94 | logarithm_glszm_SmallAreaEmphasis | 0.000 |
| 95 | logarithm_glszm_ZonePercentage | 0.000 |
| 96 | logarithm_glszm_ZoneVariance | 0.002 |
| 97 | logarithm_ngtdm_Complexity | 0.000 |
| 98 | logarithm_ngtdm_Contrast | 0.000 |
| 99 | square_firstorder_10Percentile | 0.000 |
| 100 | square_firstorder_90Percentile | 0.000 |
| 101 | square_firstorder_Energy | 0.000 |
| 102 | square_firstorder_InterquartileRange | 0.000 |
| 103 | square_firstorder_Kurtosis | 0.010 |
| 104 | square_firstorder_Maximum | 0.017 |
| 105 | square_firstorder_MeanAbsoluteDeviation | 0.000 |
| 106 | square_firstorder_Mean | 0.000 |
| 107 | square_firstorder_Median | 0.000 |
| 108 | square_firstorder_Range | 0.017 |
| 109 | square_firstorder_RobustMeanAbsoluteDeviation | 0.000 |
| 110 | square_firstorder_RootMeanSquared | 0.000 |
| 111 | square_firstorder_Skewness | 0.001 |
| 112 | square_firstorder_TotalEnergy | 0.000 |
| 113 | square_firstorder_Variance | 0.004 |
| 114 | squareroot_firstorder_10Percentile | 0.000 |
| 115 | squareroot_firstorder_90Percentile | 0.000 |
| 116 | squareroot_firstorder_Entropy | 0.009 |
| 117 | squareroot_firstorder_InterquartileRange | 0.027 |
| 118 | squareroot_firstorder_Kurtosis | 0.002 |
| 119 | squareroot_firstorder_MeanAbsoluteDeviation | 0.006 |
| 120 | squareroot_firstorder_Mean | 0.000 |
| 121 | squareroot_firstorder_Median | 0.000 |
| 122 | squareroot_firstorder_Minimum | 0.003 |
| 123 | squareroot_firstorder_RobustMeanAbsoluteDeviation | 0.018 |
| 124 | squareroot_firstorder_RootMeanSquared | 0.000 |

| | | |
|---|---|---|
| 125 | squareroot_firstorder_Uniformity | 0.007 |
| 126 | squareroot_firstorder_Variance | 0.003 |
| 127 | squareroot_glcm_ClusterProminence | 0.005 |
| 128 | squareroot_glcm_ClusterShade | 0.004 |
| 129 | squareroot_glcm_ClusterTendency | 0.005 |
| 130 | squareroot_glcm_Contrast | 0.002 |
| 131 | squareroot_glcm_Correlation | 0.005 |
| 132 | squareroot_glcm_DifferenceAverage | 0.003 |
| 133 | squareroot_glcm_DifferenceEntropy | 0.003 |
| 134 | squareroot_glcm_DifferenceVariance | 0.001 |
| 135 | squareroot_glcm_Id | 0.005 |
| 136 | squareroot_glcm_Idm | 0.005 |
| 137 | squareroot_glcm_Idmn | 0.002 |
| 138 | squareroot_glcm_Idn | 0.003 |
| 139 | squareroot_glcm_InverseVariance | 0.005 |
| 140 | squareroot_glcm_JointEnergy | 0.021 |
| 141 | squareroot_glcm_JointEntropy | 0.014 |
| 142 | squareroot_glcm_MCC | 0.033 |
| 143 | squareroot_glcm_MaximumProbability | 0.012 |
| 144 | squareroot_glcm_SumEntropy | 0.015 |
| 145 | squareroot_glcm_SumSquares | 0.003 |
| 146 | squareroot_gldm_DependenceNonUniformityNormalized | 0.008 |
| 147 | squareroot_gldm_DependenceVariance | 0.008 |
| 148 | squareroot_gldm_GrayLevelVariance | 0.003 |
| 149 | squareroot_gldm_LargeDependenceEmphasis | 0.007 |
| 150 | squareroot_gldm_LargeDependenceHighGrayLevelEmphasis | 0.009 |
| 151 | squareroot_gldm_LowGrayLevelEmphasis | 0.007 |
| 152 | squareroot_gldm_SmallDependenceEmphasis | 0.006 |
| 153 | squareroot_gldm_SmallDependenceLowGrayLevelEmphasis | 0.005 |
| 154 | squareroot_glrlm_GrayLevelNonUniformityNormalized | 0.007 |
| 155 | squareroot_glrlm_GrayLevelVariance | 0.004 |
| 156 | squareroot_glrlm_LongRunEmphasis | 0.005 |
| 157 | squareroot_glrlm_LongRunHighGrayLevelEmphasis | 0.018 |
| 158 | squareroot_glrlm_LongRunLowGrayLevelEmphasis | 0.023 |
| 159 | squareroot_glrlm_LowGrayLevelRunEmphasis | 0.009 |
| 160 | squareroot_glrlm_RunEntropy | 0.032 |
| 161 | squareroot_glrlm_RunLengthNonUniformityNormalized | 0.004 |
| 162 | squareroot_glrlm_RunPercentage | 0.003 |
| 163 | squareroot_glrlm_RunVariance | 0.004 |
| 164 | squareroot_glrlm_ShortRunEmphasis | 0.003 |
| 165 | squareroot_glrlm_ShortRunLowGrayLevelEmphasis | 0.007 |
| 166 | squareroot_glszm_GrayLevelNonUniformityNormalized | 0.035 |
| 167 | squareroot_glszm_GrayLevelVariance | 0.023 |

| | | |
|---|---|---|
| 168 | squareroot_glszm_ZonePercentage | 0.007 |
| 169 | squareroot_ngtdm_Complexity | 0.008 |
| 170 | squareroot_ngtdm_Contrast | 0.002 |
| 171 | wavelet-LLH_firstorder_Kurtosis | 0.044 |
| 172 | wavelet-LLH_firstorder_Median | 0.015 |
| 173 | wavelet-LLH_glcm_MCC | 0.039 |
| 174 | wavelet-LHL_glcm_Imc1 | 0.042 |
| 175 | wavelet-LHH_firstorder_Minimum | 0.030 |
| 176 | wavelet-LHH_glcm_Autocorrelation | 0.032 |
| 177 | wavelet-LHH_glcm_JointAverage | 0.032 |
| 178 | wavelet-LHH_glcm_MCC | 0.009 |
| 179 | wavelet-LHH_glcm_SumAverage | 0.032 |
| 180 | wavelet-LHH_gldm_HighGrayLevelEmphasis | 0.031 |
| 181 | wavelet-LHH_gldm_LargeDependenceLowGrayLevelEmphasis | 0.022 |
| 182 | wavelet-LHH_gldm_LowGrayLevelEmphasis | 0.040 |
| 183 | wavelet-LHH_gldm_SmallDependenceHighGrayLevelEmphasis | 0.039 |
| 184 | wavelet-LHH_glrlm_HighGrayLevelRunEmphasis | 0.030 |
| 185 | wavelet-LHH_glrlm_LongRunHighGrayLevelEmphasis | 0.046 |
| 186 | wavelet-LHH_glrlm_LongRunLowGrayLevelEmphasis | 0.025 |
| 187 | wavelet-LHH_glrlm_LowGrayLevelRunEmphasis | 0.045 |
| 188 | wavelet-LHH_glrlm_ShortRunHighGrayLevelEmphasis | 0.025 |
| 189 | wavelet-LHH_glszm_HighGrayLevelZoneEmphasis | 0.033 |
| 190 | wavelet-LHH_glszm_LargeAreaLowGrayLevelEmphasis | 0.045 |
| 191 | wavelet-LHH_glszm_SmallAreaHighGrayLevelEmphasis | 0.029 |
| 192 | wavelet-HLH_glcm_MCC | 0.026 |
| 193 | wavelet-LLL_firstorder_10Percentile | 0.000 |
| 194 | wavelet-LLL_firstorder_90Percentile | 0.000 |
| 195 | wavelet-LLL_firstorder_Energy | 0.011 |
| 196 | wavelet-LLL_firstorder_Kurtosis | 0.019 |
| 197 | wavelet-LLL_firstorder_Mean | 0.000 |
| 198 | wavelet-LLL_firstorder_Median | 0.000 |
| 199 | wavelet-LLL_firstorder_RootMeanSquared | 0.000 |
| 200 | wavelet-LLL_firstorder_TotalEnergy | 0.011 |
| 201 | wavelet-LLL_glcm_Idmn | 0.044 |
| 202 | wavelet-LLL_glcm_MCC | 0.037 |
| 203 | wavelet-LLL_gldm_DependenceEntropy | 0.029 |
| 204 | wavelet-LLL_glrlm_RunEntropy | 0.039 |
| 205 | wavelet-LLL_glszm_GrayLevelNonUniformityNormalized | 0.018 |
| 206 | wavelet-LLL_glszm_ZoneEntropy | 0.030 |

**Supplementary Table S2** Optimal parameters of the machine learning models

| Model | Parameter |
|---|---|
| AB | {n_estimators = 55, algorithm = 'SAMME'} |
| BAG | {n_estimators = 82, max_features = 6} |
| DT | {criterion = 'gini', max_features = 4} |
| GB | {n_estimators = 91, max_features = 1} |
| GNB | {} |
| KNN | {n_neighbors = 5, weights = 'distance'} |
| LDA | {solver = 'lsqr', shrinkage = 'auto'} |
| LR | {Cs = 10, cv = 10, penalty = 'l2', solver = 'liblinear', max_iter = 100} |
| NN | {activation = 'relu', solver = 'adam', max_iter = 2000} |
| QDA | {} |
| RF | {n_estimators = 61, max_features = 3} |
| SVM | {C = 1.2001, kernel = 'rbf', gamma = 0.0934, probability = True} |

*Abbreviations:* AB, adaptive boosting; BAG, bagging; DT, decision tree; GB, gradient boosting; GNB, Gaussian naive Bayes; KNN, k-nearest neighbors; LDA, linear discriminant analysis; LR, logistic regression; NN, neural network; QDA, quadratic discriminant analysis; RF, random forest; SVM, support vector machine.
